# Supplementary material for: From fear of infection to awareness against stigma: A mixed-methods analysis of discourses on HIV in a parliamentary context
Source: PLoS One. 2025 Oct 6;20(10):e0333537. doi: 10.1371/journal.pone.0333537 (PMC12500096; doi:10.1371/journal.pone.0333537)
Supplement: S1 Table — This checklist contains the Standard for Reporting Qualitative Research (SRQR) items. (DOCX) [file pone.0333537.s001.docx]

| **Supplementary Table S1. Standards for Reporting Qualitative Research (SRQR) checklist** | | |
| --- | --- | --- |
|  |  |  |
| **Title and abstract** | | **Page/line no(s).** |
|  | |  |
|  | **Title** - Concise description of the nature and topic of the study Identifying the study as qualitative or indicating the approach (e.g., ethnography, grounded theory) or data collection methods (e.g., interview, focus group) is recommended | Title |
|  | **Abstract** - Summary of key elements of the study using the abstract format of the intended publication; typically includes background, purpose, methods, results, and conclusions | Abstract (p. 1) |
|  |  |  |
| **Introduction** | |  |
|  | **Problem formulation** - Description and significance of the problem/phenomenon studied; review of relevant theory and empirical work; problem statement | Problem: paragraphs 8-10 of the introduction  Theory and empirical work: paragraphs 2-6, 8 |
|  | **Purpose or research questio**n - Purpose of the study and specific objectives or questions | Paragraph 12 of the introduction |
|  |  |  |
| **Methods** | |  |
|  | **Qualitative approach and research paradigm** - Qualitative approach (e.g., ethnography, grounded theory, case study, phenomenology, narrative research) and guiding theory if appropriate; identifying the research paradigm (e.g., postpositivist, constructivist/ interpretivist) is also recommended; rationale | Paragraphs of 9-11 of materials and methods |
|  | **Researcher characteristics and reflexivity** - Researchers’ characteristics that may influence the research, including personal attributes, qualifications/experience, relationship with participants, assumptions, and/or presuppositions; potential or actual interaction between researchers’ characteristics and the research questions, approach, methods, results, and/or transferability | Positionality and reflexivity statement section of materials and methods |
|  | **Context** - Setting/site and salient contextual factors; rationale | Materials and methods paragraph 1 |
|  | **Sampling strategy** - How and why research participants, documents, or events were selected; criteria for deciding when no further sampling was necessary (e.g., sampling saturation); rationale | Materials and methods paragraph 2 |
|  | **Ethical issues pertaining to human subjects** - Documentation of approval by an appropriate ethics review board and participant consent, or explanation for lack thereof; other confidentiality and data security issues | Ethics and Consent section of methods |
|  | **Data collection methods** - Types of data collected; details of data collection procedures including (as appropriate) start and stop dates of data collection and analysis, iterative process, triangulation of sources/methods, and modification of procedures in response to evolving study findings; rationale | Materials and methods paragraphs 1, 2 and 4 |
|  | **Data collection instruments and technologies** - Description of instruments (e.g., interview guides, questionnaires) and devices (e.g., audio recorders) used for data collection; if/how the instrument(s) changed over the course of the study | Materials and methods paragraph 2 |
|  | **Units of study** - Number and relevant characteristics of participants, documents, or events included in the study; level of participation (could be reported in results) | Results paragraph 1 |
|  | **Data processing** - Methods for processing data prior to and during analysis, including transcription, data entry, data management and security, verification of data integrity, data coding, and anonymization/de-identification of excerpts | NA |
|  | **Data analysis** - Process by which inferences, themes, etc., were identified and developed, including the researchers involved in data analysis; usually references a specific paradigm or approach; rationale | Materials and methods paragraphs 9-12 |
|  | **Techniques to enhance trustworthiness** - Techniques to enhance trustworthiness and credibility of data analysis (e.g., member checking, audit trail, triangulation); rationale | Materials and methods paragraph 12 |
|  |  |  |
| **Results/findings** | |  |
|  | **Synthesis and interpretation** - Main findings (e.g., interpretations, inferences, and themes); might include development of a theory or model, or integration with prior research or theory | Quantitative and qualitative sections of results |
|  | **Links to empirical data** - Evidence (e.g., quotes, field notes, text excerpts, photographs) to substantiate analytic findings | Quotes are available in the “Critical discourse analysis by parliamentary groups ideology” of results |
|  |  |  |
| **Discussion** | |  |
|  | **Integration with prior work, implications, transferability, and contribution(s) to the field -** Short summary of main findings; explanation of how findings and conclusions connect to, support, elaborate on, or challenge conclusions of earlier scholarship; discussion of scope of application/generalizability; identification of unique contribution(s) to scholarship in a discipline or field | Along the discussion summary of findings, comparison with previous evidence and theoretical framework, as well as contributions are included.  Generalizability is discussed in the strengths and limitations section |
|  | **Limitations** - Trustworthiness and limitations of findings | Strengths and limitations section of the discussion |
|  |  |  |
| **Other** | |  |
|  | **Conflicts of interest** - Potential sources of influence or perceived influence on study conduct and conclusions; how these were managed | Acknowledgements paragraph 3 |
|  | **Funding** - Sources of funding and other support; role of funders in data collection, interpretation, and reporting | Acknowledgements paragraph 4 |
